# P-566. Epidemiology of Invasive GAS Infection In Relation to CoVID-19 Era: An Epic Cosmos Study

**DOI:** 10.1093/ofid/ofaf695.781

**Published:** 2026-01-11

**Authors:** Hunter Pool, Mustafa Bakir, Collins Odhiambo

**Affiliations:** University of Illiniois College of Medicine- Peoria, Normal, IL; University of Illinois College of Medicine- Peoria, Peoria, Illinois; University of Illinois College of Medicine- Peoria, Peoria, Illinois

## Abstract

**Background:**

Invasive Group A Streptococcus (iGAS) infections have increased recently since the COVID-19 pandemic. This is seen at our institution, Children’s Hospital of Illinois, and via CDC and WHO reporting. Prior to the start of the pandemic, approximately 14,000 to 25,000 cases of iGAS disease occurred in the US each year, with approximately 1,500 to 2,300 deaths.

**Figure:**
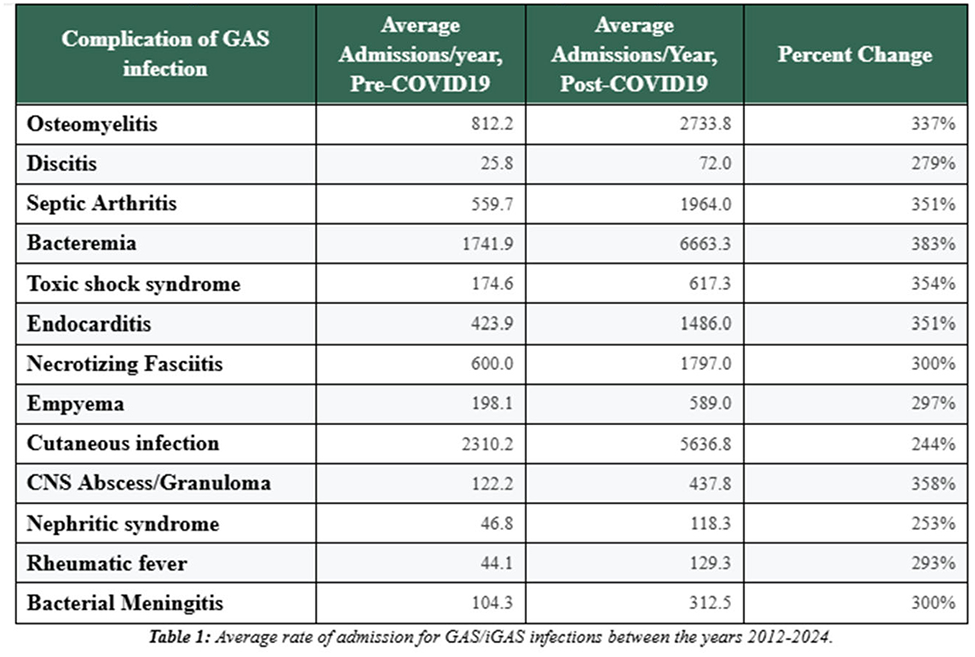
Rate of Complications of GAS/iGAS, pre vs post CoVID-19

**Figure:**
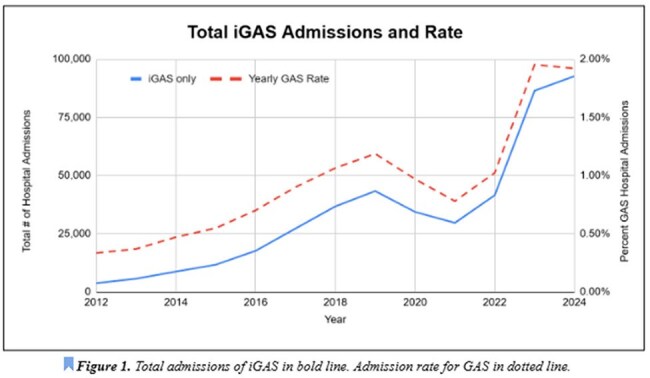
iGAS Total Admissions and Rate

**Methods:**

This study was conducted using Epic Cosmos, to obtain hospital encounter data from facilities utilizing Epic across the United States. Data was collected from 2012-2024 on all hospitalized patients. ICD codes related to iGAS infection, ICD codes for complications, age, and state of admission to iGAS infection were collected and reported.

**Figure:**
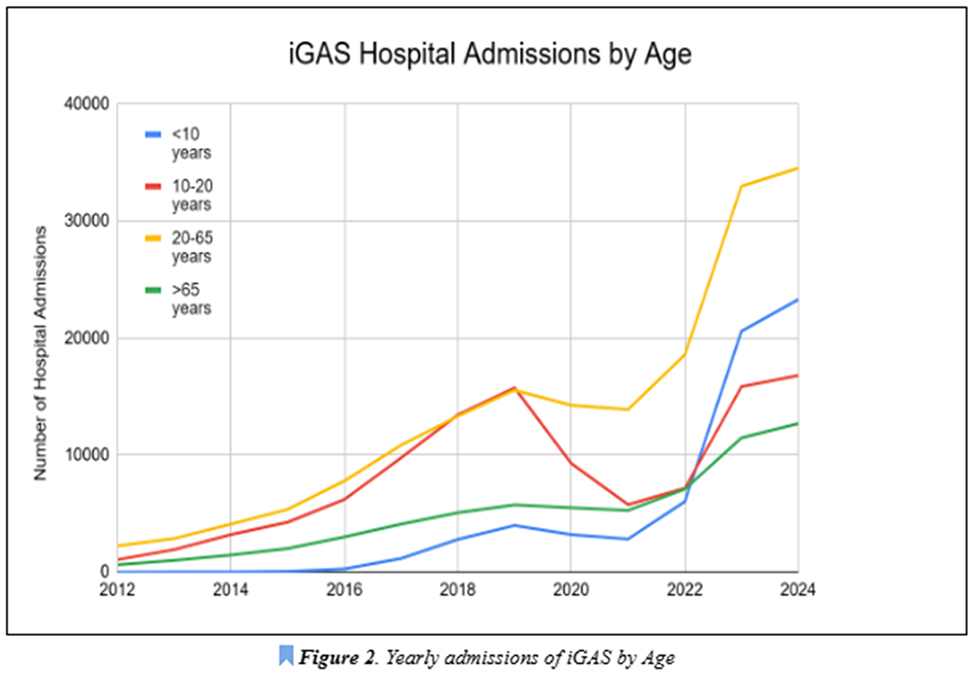
iGAS Hospital Admissions by Age

**Figure:**
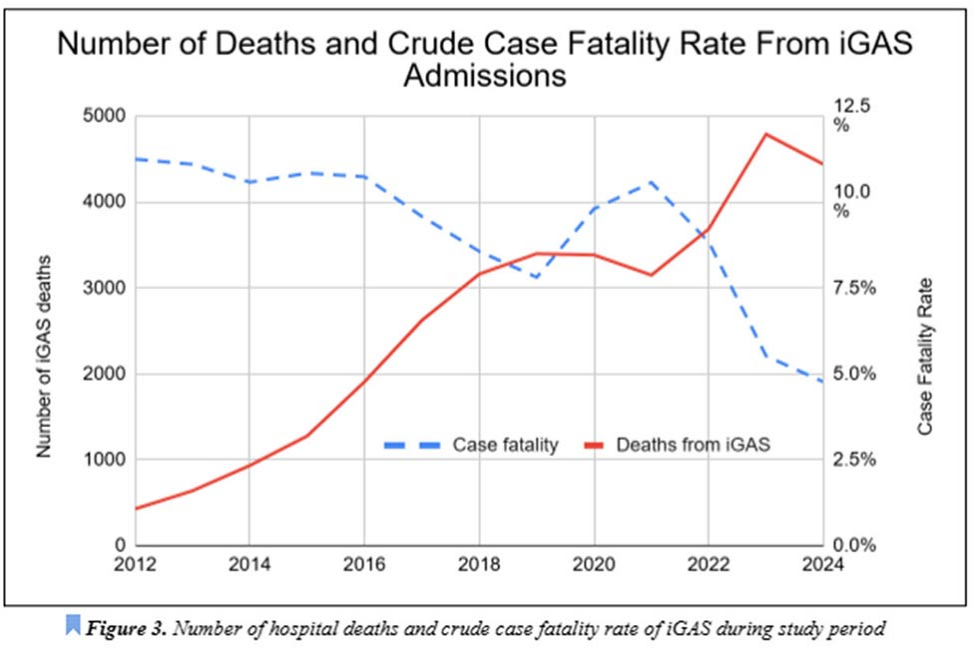
iGAS Crude Case Fatality Rate and Total Deaths

**Results:**

Data collected spans 40,166,240 hospital admissions. The average yearly admission rate pre and post pandemic is 2,553,308 and 4,296,617, respectively. Average yearly admission rate of iGAS pre and post pandemic was 21,052 and 62,608 respectively. The total number of iGAS admissions is shown to increase since the start of data collection in 2012 (Figure 1), with a steep increase in rate in 2021. There is a 177% increase in the rate of iGAS hospitalizations when comparing pre and post-COVID-19 data. Age data indicate that there is a 1,028% higher rate of iGAS hospitalizations in children less than 10 years old, but all ages show an increased rate of admission post-pandemic (Figure 2). Admission rate increased in all complications from iGAS, ranging from 244-383%, with the highest being in bacteremia (Table 1). Beyond iGAS infections, an increased rate of post Streptococcal nephritic syndromes and rheumatic fever was noted, by 253% and 293%, respectively. With regards to case fatality, the average pre and post pandemic is 10% and 7%, respectively, with a decline starting in 2021 (Figure 3). State data was extracted and showed Ohio having the highest number of pre and post-COVID iGAS admissions at 19,120 and 36,155 cases, respectively.

**Conclusion:**

This increase in admission rates of iGAS post-COVID-19 pandemic is seen across many studies, including this one. By utilizing Epic Cosmos, we can better approximate iGAS rates across the nation, given estimates of Epic utilization are around 40-50% covering approximately 300 million patients in the US.

**Disclosures:**

All Authors: No reported disclosures

